# Enablers and barriers for women with gestational diabetes mellitus to achieve optimal glycaemic control – a qualitative study using the theoretical domains framework

**DOI:** 10.1186/s12884-018-1710-8

**Published:** 2018-04-11

**Authors:** Ruth Martis, Julie Brown, Judith McAra-Couper, Caroline A. Crowther

**Affiliations:** 1 Liggins Institute, The University of Auckland, Private Bag 92019, Auckland, 1142 New Zealand; 2 Faculty of Health & Environmental Sciences, AUT, Auckland University of Technology, Private Bag 92006, Auckland, 1142 New Zealand

**Keywords:** Gestational diabetes mellitus, Pregnant women, Self-management, Dietary advice, Exercise, Health literacy, Theoretical domains framework

## Abstract

**Background:**

Glycaemic target recommendations vary widely between international professional organisations for women with gestational diabetes mellitus (GDM). Some studies have reported women’s experiences of having GDM, but little is known how this relates to their glycaemic targets. The aim of this study was to identify enablers and barriers for women with GDM to achieve optimal glycaemic control.

**Methods:**

Women with GDM were recruited from two large, geographically different, hospitals in New Zealand to participate in a semi-structured interview to explore their views and experiences focusing on enablers and barriers to achieving optimal glycaemic control. Final thematic analysis was performed using the Theoretical Domains Framework.

**Results:**

Sixty women participated in the study. Women reported a shift from their initial negative response to accepting their diagnosis but disliked the constant focus on numbers. Enablers and barriers were categorised into ten domains across the three study questions. Enablers included: the ability to attend group teaching sessions with family and hear from women who have had GDM; easy access to a diabetes dietitian with diet recommendations tailored to a woman’s context including ethnic food and financial considerations; free capillary blood glucose (CBG) monitoring equipment, health shuttles to take women to appointments; child care when attending clinic appointments; and being taught CBG testing by a community pharmacist. Barriers included: lack of health information, teaching sessions, consultations, and food diaries in a woman’s first language; long waiting times at clinic appointments; seeing a different health professional every clinic visit; inconsistent advice; no tailored physical activities assessments; not knowing where to access appropriate information on the internet; unsupportive partners, families, and workplaces; and unavailability of social media or support groups for women with GDM. Perceived judgement by others led some women only to share their GDM diagnosis with their partners. This created social isolation.

**Conclusion:**

Women with GDM report multiple enablers and barriers to achieving optimal glycaemic control. The findings of this study may assist health professionals and diabetes in pregnancy services to improve their care for women with GDM and support them to achieve optimal glycaemic control.

## Background

In New Zealand one in eleven pregnant women is diagnosed with gestational diabetes mellitus (GDM) [1]. Maternal hyperglycaemia associated with GDM is a potentially serious complication that can result in short- and long-term health risks for the woman and her baby [2–5]. Optimal blood glucose regulation within recommended glycaemic targets using lifestyle changes and/or pharmacological treatments aims to reduce or prevent the adverse outcomes associated with GDM [6–8]. A woman’s perceptions of GDM may influence whether she embraces any lifestyle changes, complies with the recommended treatment, and achieves optimal blood glucose control [9].

The New Zealand Health and Disability Commissioner has identified that consumer (a health system user) involvement is a priority in health decision making [10]. Legislation such as the Health and Disability Commissioner Act 1994 [11], and the Health and Disability Services Consumers’ Rights 1996 Code [12] support this. International organisations including Cochrane and the World Health Organisation (WHO) concur [13, 14]. They recommend that for any research involving consumers, their experiences should be investigated to support the research results [11–14].

In 2015 the National Institute for Health and Care Excellence (NICE) published an up-dated guideline for ‘Diabetes in pregnancy: management from preconception to the postnatal period’ and recommended that further robust qualitative studies were needed to explore enablers and barriers for women with GDM to maintain optimal glycaemic blood control [15]. Increased understanding of the enablers and barriers for women with GDM may help facilitate behaviour change and assist health care professionals to support women with GDM more effectively to overcome the barriers identified and support the enablers.

The use of the Theoretical Domains Framework (TDF), which informed data analysis, is an effective tool to identify enablers and barriers and to understand, inform and facilitate effective behavioural change and health service provision [16–18]. TDF was developed using an expert consensus process and validation to identify psychological and organisational theory relevant to behaviour change [16–19]. The most recent validated version of the TDF includes 14 domains and their component constructs [20] (Table 1). The TDF has been used in health care to identify factors influencing health practitioner’s clinical behaviour and behaviour change [20–22] but is increasingly being used to identify enablers and barriers for the consumer (user of health care) to understand their experiences and views to adherence of treatment and lifestyle changes [23–26].

**Table 1:** Refined Theoretical Domains Framework adapted from Cane et al. 2012 [19] and Atkins et al. 2017 [20]

Theoretical Domains	Generic Definitions	Constructs
Knowledge	An awareness of the existence of something	- Knowledge (including knowledge of condition/scientific rationale) - Procedural knowledge - Knowledge of task environment
Skills	An ability or proficiency acquired through practice	- Skills - Skills development - Competence - Ability - Interpersonal skills - Practice - Skill assessment
Social/Professional Role & Identity	A coherent set of behaviours and displayed personal qualities of an individual in a social or work setting	- Professional identity - Professional role - Social identity - Identity - Professional boundaries - Professional confidence - Group identity - Leadership - Organisational commitment
Beliefs about capabilities	Acceptance of the truth, reality or validity about an ability, talent, or facility that a person can put to constructive use	- Self-confidence - Perceived competence - Self-efficacy - Perceived behavioural control - Beliefs - Self-esteem - Empowerment - Professional confidence
Optimism	The confidence that things will happen for the best or that desired goals will be attained	- Optimism - Pessimism - Unrealistic optimism - Identity
Beliefs about consequences	Acceptance of the truth, reality, or validity about outcomes of a behaviour in a given situation	- Beliefs - Outcome expectancies - Characteristics of outcome expectancies - Anticipated regret - Consequents
Reinforcement	Increasing the probability of a response by arranging a dependent relationship, or contingency, between the response and a given stimulus	- Rewards (proximal/distal, valued/not valued, probable/improbable) - Incentives - Punishment - Consequents - Reinforcement - Contingencies - Sanctions
Intentions	A conscious decision to perform a behavior or a resolve to act in a certain way	- Stability of intentions - Stages of change model - Transtheoretical model and stages of change
Goals	Mental representations of outcomes or end states that an individual wants to achieve	- Goals (distal/proximal) - Goal priority - Goal/target setting - Goals (autonomous/controlled) - Action planning - Implementation intention
Memory, attention, and decision processes	The ability to retain information, focus selectively on aspects of the environment and choose between two or more alternatives	- Memory - Attention - Attention control - Decision making - Cognitive overload/tiredness
Environmental context and resources	Any circumstance of a person’s situation or environment that discourages or encourages the development of skills and abilities, independence, social competence, and adaptive behaviour	- Environmental stressors - Resources/material resources - Organisational culture/climate - Salient events/critical incidents - Person × environment interaction - Barriers and facilitators
Social influences	Those interpersonal processes that can cause individuals to change their thoughts, feelings or behaviours	- Social pressure - Social norms - Group conformity - Social comparisons - Group norms - Social support - Power - Intergroup conflict - Alienation - Group identity - Modelling
Emotion	A complex reaction pattern, involving experiential, behavioural and physiological elements, by which the individual attempts to deal with a personally significant matter or event	- Fear - Anxiety - Affect - Stress - Depression - Positive/negative affect - Burn-out
Behavioural regulation	Anything aimed at managing or changing objectively observed or measured actions	- Self-monitoring - Breaking habit - Action planning

The aims of this study were to explore the views and experiences of women with GDM with a focus on enablers and barriers to achieving optimal CBG control. Initially women were asked about how they felt and reacted when they were first diagnosed with GDM and if these impressions changed over time. To achieve the aims of the study, three broad questions were explored with participating women:

1. What is it like for a woman to monitor their CBG concentrations?
2. What affects a woman’s capillary CBG concentrations and how does she maintain optimal CBG control with this knowledge?
3. What support have women found helpful/not helpful in learning about and maintaining optimal CBG control?

## Methods

### Study design and procedure

This was a qualitative descriptive study and thematic content analysis as informed by Braun and Clarke and the Theoretical Domains Framework was used to analyse the data [16, 20, 27, 28]. Semi-structured interviews enabled women with GDM to express their views and experiences in their own words [29, 30]. Women could choose to be interviewed face-to-face, or to be telephoned. Women were made aware that the interview was not an assessment of their knowledge about GDM and that they could stop the interview at any time. They were advised that all their information would be kept confidential. All women chose a pseudonym at the end of the interview for de-identifying their data and for use when disseminating the results.

This qualitative study was nested within the TARGET Trial (Optimal Glycaemic Targets for Gestational Diabetes), a stepped wedge randomised controlled trial (Australian New Zealand Trial Registry: ACTRN12615000282583), which is assessing less tight and tighter glycaemic targets for women with GDM and the effect on maternal and perinatal morbidities. The study was approved by the New Zealand Health and Disability Ethics committee (HDEC) Ref. 14/NTA/163, research registration number 1965. Locality agreements were obtained from Canterbury and Counties Manukau District Health Boards (DHB).

### Study setting

Two New Zealand tertiary hospitals participated, one from the South Island (Canterbury DHB)) and one from the North Island (Counties Manukau DHB). Hospital policies differed for glycaemic targets and testing of capillary blood glucose (CBG). During the study, Canterbury DHB moved from initially less tight glycaemic targets (fasting blood glucose < 5.5 mmol/L; 1-h postprandial < 8.0 mmol/L; and 2-h postprandial < 7.0 mmol/L) to tighter targets (fasting blood glucose ≤5.0 mmol/L; 1-h postprandial ≤7.4 mmol/L; and 2-h postprandial ≤6.7 mmol/L). Women were asked to test their CBG at one-hour postprandial. Counties Manukau DHB used tighter glycaemic targets during the study (fasting blood glucose ≤5.0 mmol/L; 1-h postprandial ≤7.4 mmol/L; and 2-h postprandial ≤6.7 mmol/L) and women were asked to test their CBG two-hours postprandial.

### Study participants

Women with GDM were eligible to participate if they had not yet given birth, had a singleton pregnancy, were able to communicate in English and had been self-monitoring their CBG concentrations for at least two weeks. All women with GDM recruited between August 2016 to February 2017 for the TARGET Trial at Canterbury and Counties Manukau DHB were sent an email invitation to consider participation in this nested study with a participant information sheet and consent form attached. Eligible women who wished to participate signed a consent form for this study.

### Study materials

A question guide to facilitate the semi-structured interview was developed and pilot tested with three women who had GDM. This resulted in the addition of one question about hunger and adding the request for a pseudonym for identification rather than only a number to identify the data of participants. The data from these three women involved in piloting the question guide were included in the analyses. If women needed further guidance to share their thoughts, the question guide listed prompts and sub-questions for each broad question.

### The semi-structured interview

One researcher (RM), with facilitating skills, conducted all the interviews over a six months’ time period (August 2016 to February 2017). The woman’s choice directed the place and timing of the interview. Consequently, face-to-face interviews were conducted at a variety of settings including a woman’s home, work place, botanical gardens, cafés, on farms and hospital sites. No time constraints were applied for the interviews with most lasting about 40 min.

### Data collection and analysis

All interviews were recorded using a digital recorder and were transcribed verbatim using Microsoft 2010 by independent transcribers, who had signed a confidentiality agreement. The transcripts were verified by the researcher (RM) and entered NVivo11 for windows [31] for data management and analysis. Thematic content analysis was conducted initially using an inductive approach [28] where transcripts were read and re-read in full for familiarisation with the data and analysed using open coding techniques assigning a code to each meaningful segment of text. As the open codes became saturated, a list of specific themes was generated, compared and categorised to broader overarching themes, following Braun’s steps 1–5 [28] (Table 2). This was followed by a deductive approach assigning the themes with meaningful text to one or more of the 14 theoretical domains reflected in the Theoretical Domain’s Framework [16, 20] (Table 1).

**Table 2:** Braun’s (2006) Thematic Analysis Approach adapted from Braun et al. 2006 [28]

Steps	Content
1. Familiarisation with the data	Reading and re-reading the data, to become immersed and intimately familiar with its content
2. Coding	Generating succinct labels (codes) that identify important features of the data that might be relevant to answering the research question. It involves coding the entire dataset, and after that, collating all the codes and all relevant data extracts, together for later stages of analysis.
3. Searching for themes	Examining the codes and collated data to identify significant broader patterns of meaning (potential themes). It then involves collating data relevant to each candidate theme, so that you can work with the data and review the viability of each candidate theme.
4. Reviewing themes	Checking the candidate themes against the dataset, to determine that they tell a convincing story of the data, and one that answers the research question. In this phase, themes are typically refined, which sometimes involves them being split, combined, or discarded.
5. Defining and naming themes	Developing a detailed analysis of each theme, working out the scope and focus of each theme, determining the ‘story’ of each. It also involves deciding on an informative name for each theme.
6. Writing up	Weaving together the analytic narrative and data extracts and contextualising the analysis in relation to existing literature.

Two researchers (RM and JB) coded and classified the data and consulted with the other authors (JMC and CAC) to discuss and revise synthesising the text into the final behavioural domains with enablers and barriers identification for aspects of optimal glycaemic control. Where text fitted into multiple domains, two researchers (RM and JB) discussed and decided which text should be coded into the domain that best reflects the key theme [19] and whether a statement represented a barrier or enabler to achieving optimal glycaemic control. Reporting of this study was based on the COREQ (**Co**nsolidated Criteria for **Re**porting **Q**ualitative Research) checklist [32].

## Results

**Fig. 1:**
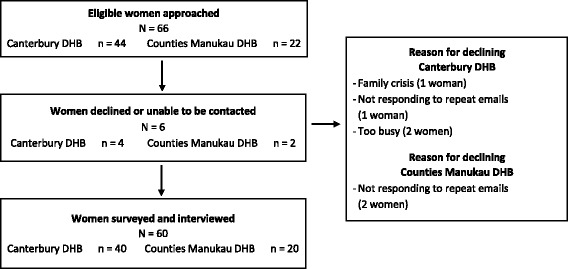
Flowchart of recruitment

During the study period, sixty-six eligible women with GDM consecutively recruited to the TARGET Trial were approached. Six women declined to be part of this study because they were too busy, having a family crisis or did not respond to the email invitation (Fig. 1). Twenty women with GDM were recruited from the Counties Manukau DHB site and 40 women with GDM from Canterbury DHB site, giving a total of 60 participants. The sociodemographic characteristics of the women who participated are reflective of a cross section of the demographics of New Zealand’s pregnant population [33–35] (Table 3). Data were analysed and coded from 858 transcribed pages (249,692 words).

**Table 3:** Demographic characteristics of women who participated in the survey

Characteristics	Women total *n* = 60 (%)
Age (years)^a^	33 (±4.5)
Primigravida (G_1_P_0_)	27 (45)
*BMI category* ^b^	
- Normal	21 (35)
- Overweight	11 (18.3)
- Obese (Class I)	11 (18.3)
- Obese (Class II)	8 (13.3)
- Obese (Class II)	9 (15)
- Total obese	28 (46.6)
*Ethnicity* ^c^	
- European	24 (40)
- Māori	6 (10)
- Asian	22 (36.7)
- Pacific Peoples	7 (11.6)
- MELAA	1 (1.7)
*Highest educational qualifications after leaving school* ^d^	
1. No qualification	3 (5)
2. Level 1 certificate	2 (3.3)
3. Level 2 certificate	4 (6.7)
4. Level 3 certificate	6 (10)
5. Level 4 certificate	4 (6.7)
6. Level 5 and level 6 Diploma	13 (21.7)
7. Bachelor degree and level 7 qualification	25 (41.6)
8. Post-graduate and honours degree	1 (1.7)
9. Master degree	2 (3.3)
New Zealand Deprivation index^e^	
- 1 (least deprived)	8 (13.5)
- 2	5 (8.4)
- 3	5 (8.4)
- 4	10 (16.7)
- 5	7 (11.8)
- 6	2 (3.4)
- 7	5 (8.5)
- 8	6 (10)
- 9	5 (8.7)
- 10 (most deprived)	6 (10)
*Lead Maternity Carer (LMC)* ^f^	
- Midwife	55 (91.7)
- Obstetrician	1 (1.7)
- Hospital Team	4 (6.7)
*Health history*	
Gestational age at GDM diagnosis (weeks)^a^	27.8 (±2.0)
Previous GDM	10 (16.7)
Previous hypertension	2 (3.3)
Current hypertension	3 (5)
Family history of hypertension	24 (40)
Family history of diabetes	27 (45)
Current smoker	3 (15)
*Capillary blood glucose testing (CBG)*	
Weeks of self-testing capillary blood glucose at interview^a^	6.8 (±2.3)
Daily self-testing CBG: four times (Before breakfast, after breakfast, after lunch and after dinner)	32 (53)
Daily self-testing CBG: six times (Before and after breakfast, lunch and dinner)	28 (47)
*Current treatment*	
- Diet only	18 (30)
- Insulin and diet	13 (21.7)
- Metformin and diet	17 (28.3)
- Insulin, Metformin and diet	12 (20)
*Interview type*	
Face-to-face interview	34 (57)
Phone interview	26 (43)

Figures are numbers and percentages

^a^Mean and standard deviation

^b^BMI categories: Underweight < 18.50; Normal range: ≥ 18.55–24.99; Overweight: ≥ 25.00–29.99; Obese (Class I) ≥ 30.00–34.99; Obese (Class II): Severe obese ≥35.00–39.99; Obese (Class II): Morbid obese: ≥ 40.00 (according to WHO and Ministry of Health categories) [44, 45]

^c^as categorised by New Zealand government statistics groups for major ethnic groups. MELAA is an acronym for Middle Eastern/Latin American/African. http://www.stats.govt.nz/Census/2013-census/profile-and-summary-reports/infographic-culture-identity.aspx

^d^as categorised by New Zealand government statistics groups. http://archive.stats.govt.nz/?_ga=2.86002648.1123263351.1521524783-1632759419.1521524783

^e^as categorised by New Zealand 2013 Deprivation Index, University of Otago, Department of Public Health. *Deprivation score was unknown for one woman, as her address had no meshblock listed*

Http://www.otago.ac.nz/wellington/departments/publichealth/research/hirp/otago020194.html

^f^A Lead Maternity Carer (LMC) in New Zealand provides lead maternity care (is in charge). This can be either a Midwife, Obstetrician, or GP. https://www.midwife.org.nz/in-new-zealand/contexts-for-practice

Women were diagnosed with GDM at a mean gestational age of 27.8 weeks (standard deviation (SD) ± 2.0. Ten women (16.7%) reported having GDM in a previous pregnancy and twenty-seven (45%) women reported a family history of diabetes (Table 3). When interviewed, the women had been checking their daily CBG for an average of 6.8 ± 2.3 weeks (Table 3). Twenty-eight women (47%) were checking their daily CBG concentration six times (before and after breakfast, lunch and dinner) and thirty-two women (53%) were checking CBG concentrations four times a day (before breakfast and after breakfast, lunch and dinner) (Table 3). Almost a third of women (18, 30%) were treated with diet alone. Thirteen (21.7%) women were treated with subcutaneous insulin for their GDM, 17 (28.3%) women with metformin and 12 (20%) women were treated with insulin and metformin (Table 3). For the interview 34 (57%) women chose to be interviewed face-to-face and 26 (43%) women by telephone (Table 3).

### Women’s initial response to being diagnosed with GDM

As an introduction to the interview, women were asked how they responded when diagnosed with GDM and if that response changed over time. This enabled women to share their emotions and thoughts about GDM, to recognise how far they had come on their journey with GDM and provided an effective platform for discussing enablers and barriers to achieving optimal glycaemic control [36]. Over a third of the women described their initial response as being shocked (21, 35%).

*“Shocked, I don’t feel like I have diabetes, as I feel normal and okay” (Belle 19A).*

Seven (11.7%) women described it as unexpected, while five (8.3%) women felt okay about the diagnosis.

*“The initial gut reaction is like, oh my God, I did not expect this and what does this mean for my baby?” (Karen 09A).*

*“I felt okay, because I know lots of Asian people, my friends around, they are pregnant. And a lot of Asian women they very, very easily get diabetes, pregnancy diabetes. So, I am prepared. I am okay” (Casey 01A).*

The remainder of women described their initial response as being disappointed (4, 6.7%), gutted (3, 5%), annoyed (3, 5%), upset (3, 5%), guilty (3, 5%), devastated (3, 5%), defeated (2, 3.3%), freaked out (2, 3.3%), angry (2, 3.3%) miffed (1, 1.7%) and heart-broken (1, 1.7%).

*“I think disappointing, because my diet’s pretty clean anyway. In that sense, it was disappointing” (Sian 11A).*

*“Can’t be true, gutted, made them do another test, otherwise I would not do the treatment” (Larissa 01B).*

### Women’s response to living with GDM at the time of the interview

Women at the time of the interview had been living with GDM for an average of 6.8 ± 2.3 weeks (Table 3). Most of the women commented that they had moved on from their initial response (49, 81.7%). They accepted the diagnosis as ‘okay’ because it would only last a finite time, but did not like the focus on the numbers of their glycaemic targets, their CBG results, weight gain and 90th percentile of fetal growth.

*“It’s hard because we have to change our routine, we have to change our food patterns and all those sort of things, changing our life to be frank, but when it comes to the reality, that makes you know, a huge difference, in our life, so it’s a big change, a big challenge but we have to accept it, even though the numbers run my life but we have to do the things. The other good thing after my delivery, it will go away” (Anna 07).*

*“It’s quite overwhelming in the beginning you kind of realise now that it’s not as big it kind of first seems. You just kind of adjust to it I guess and then its ok, always have to keep a look out for the numbers though” (Collette 09B).*

### Theoretical domains framework – Enablers and barriers

Following Braun’s [28] (Table 2) thematic content analysis, the emerging themes were categorised into the TDF domains assigning the themes with meaningful text to one or more of the 14 theoretical domains [16, 20] (Table 1) for enablers and barriers identification. The results are reported for each of the study questions. The 10 categorised domains, their definitions and identified enablers and barriers from women with GDM are listed in Tables 4, 5 and 6.

**Table 4:** Enablers and Barriers for women with GDM to monitor their CBG concentration

Domains and Definitions	Enablers	Barriers
Knowledge Refers to a woman knowing her glycaemic targets and procedural knowledge of how to test accurately	Glycaemic targets on: - sticker on the recording booklet - post-it notes on work computer - mobile phone notebook - visual step-by-step pamphlet - list how to perform CBG testing	- different glycaemic targets to previous pregnancy - unable to read the ‘how to do it list’ in first language - no visual images of how to perform CBG testing
Skills Refers to a woman’s ability to perform the CBG testing, working the glucometer correctly and documenting results and completing a food diary	Techniques for CBG flow: - alternating warm fingers & hands - not using soap - pricking on side of finger pads Food diary documenting	- no apps available for recording CBG results - food diary writing space too small - food diary not in first language - not knowing how to go back on glucometer
Beliefs about capabilities Refers to a woman’s beliefs about her capability to perform, control and monitor her CBG concentration	- can-do attitude - perceived control of GDM - in control of CBG testing - capable of interpreting CBG results and adjusting food intake	- can’t-do it attitude, too difficult - belief that it is not necessary to test regularly - perceived lack of control
Beliefs about consequences Refers to a woman’s expectations about optimal CBG control	Anticipated positive consequences: - adhering to glycaemic targets will control GDM - secure healthy future for the baby - baby will be a normal size - belief future health will be better - belief family health will be better	Anticipated negative consequences: - fingerpicks damage finger pads, too difficult to play the piano or guitar - testing and controlling CBG did not work last time
Memory, attention, and decision process Refers to a woman’s ability to remember when and decide where, to perform CBG testing	- mobile phone alarm reminder - setting timer on microwave - dedicated bag ready access to glucose testing equipment - able to decide where to do CBG testing	- forgetful - no reminder plan in place - unable to think outside the square - concern for doing CBG testing outside the home
Environmental context and resources Refers to a woman’s access to equipment and to a health professional when unsure about results	- free resources for CBG testing - phone access to diabetes midwife - booklet fits into glucometer bag - pharmacist teaching CBG testing - group teaching sessions for learning CBG testing	- costs of resources needed for CBG testing - no phone access to diabetes health professionals - booklet too big for glucometer bag - health professional not believing results
Social influences Refers to a woman’s social interactions for CBG monitoring and maintaining optimal CBG control	- supportive and engaged social interactions - do it wherever, no concern - work colleagues remind them - provide healthy food at work	- social pressure and loss of choice - worried about performing CBG testing in public, being judged - being told to leave restaurant for CBG testing - work demands, meetings, unable to stop work for CBG testing
Emotion Refers to a woman’s reaction/feelings to monitoring and maintaining her CBG concentrations	- privilege to have been diagnosed - enabled learning a new skill that directed positive lifestyle changes - fun doing everyone’s CBG level - not as painful as anticipated	- anxiety, scared, needle phobia - stress to remember doing CBG testing, - feeling guilty when forgotten - focus on numbers not the woman - not enjoying reading
Behavioural regulation Refers to a woman’s focus on self-monitoring effectively and planning how to incorporate this into her daily life	- action plan to monitor CBG - motivated by the baby to monitor CBG regularly - documenting honestly - sharing on social media glycaemic target achievements

**Table 5:** Enablers and barriers for women with GDM understanding what effects their CBG concentrations

Domains and Definitions	Enablers	Barriers
Knowledge Refers to a woman’s understanding of what affects her CBG concentrations	- understanding the difference between carbohydrates, proteins, and fats - ability to read and comprehend food labels - able to understand how physical activity or inactivity affects their CBG concentrations	- lack of understanding which foods and exercises raise the CBG concentrations - not knowing how to read food labels - knowing how to increase insulin to eat favourite sweets
Belief about consequences Refers to a woman’s expectations about what affects her CBG concentration	- eating the same food every day for optimal glycaemic control - using commercially available, pre-assembled ready for cooking, health food bags for optimal glycaemic control - hearing other women’s stories encourages anticipated regret - regular activities easy to incorporate into daily life and ensures healthy baby	- belief only medication controls CBG concentrations - belief that exercises have no effect on CBG concentrations - belief that physical activity can cause pre-term labour
Environmental context and resources Refers to a woman’s access to food, exercise equipment and health professionals	- access to dietitian and group sessions - food diary and discussion - food costs are less (no fast foods) - vegetable garden - recipes on social media - identifying food in pantry which are suitable with stickers - being organised - appropriate food available when not at home - access to exercise equipment (home bicycle, tread mill) - family and children creating motivating resources	- dietetic service unavailable - transport and time issues - not documenting a food diary or not knowing about it - health professionals do not discuss content of food diary - food is more expensive (fruit, special bread) - no ethnic food options included - unavailable professional assessment for exercise/physical activities - easy access to sugary food and drinks
Emotions Refers to a woman’s reaction/feelings to what affects her CBG concentrations	- excited to understand the link between food and exercise and CBG concentrations	- stressed about trying hard but not able to achieve optimal CBG concentrations - feeling hungry most of the time
Behavioural regulation Refers to a woman’s focus on self-monitoring effective food intake and exercise and planning how to incorporate this into daily life	- self-monitoring with food diary - developing an activity diary - calling exercise physical activity - calling diet food intake, or what to eat - action plan for physical activities - creatively incorporating family exercises affecting CBG concentrations - family and children creating motivating resources together	- dislike of exercises - medication and food is enough to maintain CBG levels - stress or excitement increases CBG concentrations, too hard to control

**Table 6:** Enablers and barriers of support for women with GDM about maintaining optimal CBG control?

Domains and Definitions	Enablers	Barriers
Beliefs about consequences Refers to a woman’s expectation to sharing her diagnosis of GDM with others	- telling others about GDM diagnosis gains valuable support	Not telling others about GDM diagnosis because: - concern for other family members - being judged by family, friends and work colleagues - being told what and what not to eat
Reinforcement Refers to a woman’s ability to reinforce skills and coping strategies for self-support in maintaining optimal glycaemic control	- continuing with food diary, feeling better - photos of food eaten instead of written food dairy - self-rewards with non-food items - documenting CBG results - activities connected with family fun	
Environmental context and resources Refers to a woman’s ability to have access to learning resources and professional services for optimal glycaemic control	- written information in first language - visual information - informative websites - partner and extended family able to attend teaching or clinic sessions - work colleagues enquiring and providing healthy food options - efficient clinic appointment system - health professional phone support - free health shuttle for appointments - hospital crèche - stickers with healthy GDM messages encourages adherence to healthy food and exercises	- health professional impatient - health professionals inconsistent with advice - not seeing the same health professional twice - long waiting times at clinic - not taught in first language - unable to write the food dairy in first language - no visual information available - website information random and scary - poor parking facilities - no transport available - unable to pay for petrol - no child care support - restaurants unable provide an ingredients list for meals - partner and extended family provide unhealthy meals
Social influences Refers to a woman’s access to social interaction to learning/reinforcing optimal glycaemic control	- social media (Facebook) - sharing recipes - group teaching - meeting other women with GDM - partner, family, and friend’s interest - work colleagues support	- unsupportive family members and workplaces - no social media groups or support groups in NZ - not knowing anyone with GDM - unable to perform testing in public - told what to eat by family members

### What is it like for a woman to monitor her CBG concentrations?

The themes emerging from the interviews from women with GDM for the first research question ‘What is it like for a woman to monitor her CBG concentrations?’ were categorised within nine out of the possible fourteen TDF domains. These were: Knowledge, Skills, Beliefs about capabilities, Beliefs about consequences, Memory, attention and decision process, Environmental context and resources, Social influences, Emotion and Behavioural regulation (Table 4). The domains represented most strongly in the interviews in the context of this question were: Beliefs about capabilities, Social influences and Emotions.

### Knowledge

The domain ‘Knowledge’ in the context of monitoring optimal CBG control refers to a woman knowing her glycaemic targets and procedural knowledge of how to test accurately. Nearly all women knew their glycaemic targets and most understood the importance of adhering to them, enabling the process of performing routinely CBG testing. Enablers to assist this knowledge were identified as having information aids, such as stickers at the front of the recording booklet displaying the glycaemic targets, post-it notes for a work computer and glycaemic targets recorded on their mobile phone. Using a visual step-by-step pamphlet to ensure correctly obtaining capillary blood for glucose testing enabled procedural knowledge retention.

*“I actually understood why I had to do this air tight control, so I do it. The sticker on the booklet reminds me of my numbers and the booklet in glucometer with pictures reminds me what to know” (Erin 04B).*

Women reported barriers as being confused by different glycaemic targets compared to their last pregnancy with GDM and information that would have aided their procedural knowledge not being provided in enough visual detail or the information was not in their first language.

*“The consultant gave me something that I haven’t looked at, but it was ‘I quit sugar’ and I wouldn’t recommend that, as it sounded like sugar is poison and all this kind of stuff. Pictures would be so much better” (Alice 10A).*

*“I think it’s important to give us something to take away, and some bullet points or pictures, now that you are diagnosed, these things you need to do, why we are doing it and in the right language” (Christina 15A).*

### Skills

The domain ‘Skills’ refers to a woman’s ability to perform the CBG testing, working the glucometer correctly and documenting results and completing a food diary. Women identified various effective techniques to enable good capillary blood flow and reducing discomfort. This included not using soap for washing hands (as a belief that soap contains sugar), pricking on the edge of the finger pads, placing sufficient pressure on finger pads, wiping the first blood spot off and alternating of warm fingers and hands. Keeping a food diary helped women to make a connection between what they ate and what the test results meant, encouraging the up-keep of both regular accurate testing and recording in the food diary.

*“Just pressing your fingers firmly against the end of the pricky thing on the side, because no one wants to do it twice. Next time either side of the next finger and then keep going to the next finger, all makes it less painful and better blood drops” (Toni 03B).*

Barriers included having a needle phobia and women feeling frustrated that a phone app for recording CBG results was not available or not being able to document into an electronically food diary. Over half of the women wanted instructions for CBG testing in their first language, the opportunity to record their food diary in their first language and more writing space in the hard copy food diary. Some women did not know how to go back on the glucometer to check their previous results. All these barriers prevented women from either mastering or performing regular glucose testing and recording their food intake.

“*To be honest, the diabetes books are quite small to write in what you are eating and that can be off putting, for me I found anyway. It would so much better to have everything on my phone, like a phone app for the blood sugars and like a kind of electronic diary, everything else is on my phone, then I would do it more regularly I think” (Janet 07A).*

*“I think they give you a lot of information that.... I mean it’s good to have. But then again, yeah, I’m probably not much of a reader. I just like to speak to thing, maybe give me a YouTube clip [link], and have picture that remind me how to, that would definitely help especially if it’s your first language” (Larissa 01B).*

### Beliefs about capabilities

‘Belief about capabilities’ refers to a woman’s belief about her capability to perform, control and monitor her CBG concentration. The most common reported constructs were self-confidence and having control to help women to do their CBG testing without concerns, alter their food intake accordingly and developed a ‘can-do attitude’. Barriers were identified as having a ‘can’t-do’ attitude, believing that it is not necessary to test their CBG concentrations regularly and a feeling of not being in control.

*“The dietician and the doctor were very impressed with my numbers, and that made me feel amazing and proud, and chuffed with myself. I can do this” (Anneri 14B).*

*“It’s too hard, I can’t do it” (Yoko 15B).*

Women who had firm beliefs about their capability reported how they were not concerned to do the CBG testing in public or at work and were highly motivated to do the testing at the appropriate times, even if it meant interrupting what they were doing.

*“I am good at this, do it always on time. I do it when I am commuting on the bus or when I am attending mass. Even though I am in the middle of kneeling and everyone is quiet, I will just quickly get out my kit and quickly prick myself even though it may make some noise” (Karroll 05B).*

Women with uncertain beliefs about their capabilities became increasingly scared to do their CBG testing. They had to repeat tests more often, as there was either not enough blood for the test or the glucometer would show error messages, which led them to test less often than recommended, or not at all. Women’s feelings voiced of not being in control, as the CBG concentrations directed their food, exercise and/or medication intake, contributed to low self-belief in their capabilities.

*“Yes, I get frustrated with it and then the glucometer does not work. Yes, I have my days where I’m tired and I’m sick of it, and belief I can’t do it. I don’t do my blood tests then, and I don’t manage my food. It just runs my life” (Karrena 17B).*

### Beliefs about consequences

This domain refers to a woman’s expectations about optimal CBG control. Participating women reported that anticipated positive or negative consequences strongly influenced their actions; whether they tested their CBG concentrations, adhered to the glycaemic targets, changed their food intake and physical activities, or took their medication. This domain was represented strongly throughout the interviews.

*“They did tell us like that if mums are not taking care, there may be a chance for the baby to have the diabetes when the baby is a teenager or when it is little, that was a good thing, that is the one reason which I’m more careful, which I don’t want to give anything to my kids which is from me you know, whatever the life brings to them that’s their luck you know, but I don’t want to give anything from me to my next generation, so you know, if I can be more careful about that then I have to, totally changed everything and never forget to do blood sugars”(Anna 07).*

*“Well, can I play the guitar with so many holes in my fingers? Who wants that? So, pricking only alternative days and not on my left hand is sort of ok, but if I have to play in church, I don’t do it the week before” (Yasmin 01).*

### Memory, attention, and decision process

This domain refers to a woman’s ability to remember when, and decide where, to perform CBG testing. Women identified memory aids, such as alarms on mobile phones, setting a timer and having a dedicated bag for all the CBG testing equipment to aid their memory and the decision process of performing the test, regardless of where they were. Forgetfulness was identified as a perceived barrier for doing regular CBG testing, in particular when away from their home, causing considerable frustration and anger for some women.

*“Yeah husband reminds me at night most of the time (Amali 16). I get my partner to ring me and then do it. A couple of times when I’ve been driving I did it while I was driving” (Angela 15). “I keep my alarm on the phone, as otherwise you know, I can’t remember the particular time” (Hana 11B). “Just put an alarm in my head and watch my clock every couple of hours” (Neethu 02).*

*“I do it anywhere… And they will ask me what are you doing? And that is the time I start talking to them about gestational diabetes and I say, ‘you know I have gestational diabetes and I have to do this’. And then about at the same time I am like a tool for everybody to find out about diabetes and they learn about it” (Karroll 05B).*

*I tend to stress about it for the first half an hour after a meal, that I’ve got to remember, and then it just slips your mind some days, so frustrating (Erin 18B).*

### Environmental context and resources

‘Environmental context and resources’ refers to a woman’s access to equipment and to a health professional when unsure about results. The most commonly reported barrier was the cost of resources, no phone access to a health professional when the woman was unsure about results and health professionals not believing the women’s documented CBG results. Different sizes of CBG recording booklets were either experienced as enablers or barriers. Some women found it frustrating that their CBG recording booklet did not fit into the glucometer bag, which meant it had to be carried separately. This meant for some women CBG results were not recorded when outside their homes.

In New Zealand women receive a free glucometer, blood lancets, and testing strips from the diabetes in pregnancy services at their local hospital or they are given a prescription for these resources to be picked up from their local pharmacy. While some women could pay the costs of the prescription fee, some women found it too difficult over time and then did not continue CBG testing.

*“It’s definitely more expensive … and then prescriptions fees for the testing bits. It all adds up and you want to be sure it’s worth it. Some weeks it is not” (Jean 16B).*

*“Yeah, like insinuating that I eat overnight, because my levels are high in the morning, like no, I am busy sleeping actually, but yeah that I struggled with, not being believed by the diabetes consultant. Why should I continue testing then?” (Alice 10A).*

### Social influences

In the context of this study the domain ‘social influences’ refers to a woman’s social interactions for CBG monitoring and maintaining optimal CBG control. Engaged social interactions, such as work colleagues asking after CBG concentrations and reminding women to do their testing, as well as providing healthy food choices and stopping meetings to provide opportunity for the women to do their testing were enablers. Barriers were being told to leave the restaurant (or other public places) when performing CBG testing and being unable to stop work for the testing. Women working as managers, bus drivers, factory workers, nurses and doctors found it difficult to adhere to the post-prandial timeframes for CBG testing, as there was often little opportunity to stop their work.

*“Well, at work they gave me private corner to do it [CBG testing] and they are really interested what my levels are. My colleagues remind me. So helpful” (Yoko 15B).*

*“I feel bad if I don’t do it, but yeah it’s usually as I’ve just been somewhere where I feel I couldn’t do it, or I can’t stop at work, especially now that I had the experience of being told to leave the restaurant and they think you are a druggie scum bag” (J.M.T.J.M.P. 14).*

### Emotion

The domain ‘Emotion’ in this study refers to a woman’s reaction and feelings to monitoring and maintaining her CBG concentrations. Some women felt it was a privilege to have been diagnosed with GDM as it meant they learnt new skills that directed positive lifestyle changes. The sense of achievements in mastering CBG collection and staying within recommended glycaemic targets enabled optimal glucose monitoring. This led to testing family and friends without understanding that this would be recorded on the glucometer as their results.

*“It’s been a good adjustment, kind of a joy, I learnt how to test blood sugars and I am living healthy, it’s kind of like a good stepping stone to continue that healthy life. It’s kind of giving you this mirror glass into the future that you could have diabetes in the future (Esther 07B). I'm brave, I never forget to do the pricks” (Raynia 09).*

Barriers included emotions of stress and being scared to do the capillary testing at the appropriate times, especially where a needle phobia existed. The constant focus on the numbers discouraged some women from performing regular CBG testing.

*“Ah yes, I am scared, first of all ‘cause I hate needles. One thing, it should be different that putting like, when we test our diabetes, the needle we put in our fingers, it’s very painful, like all my fingers have holes, because every day I prick and then I stop. So, there should be different type of thing we can measure the diabetes” (Shairin 11).*

*“Um, I guess, having had that experience before as part of my medical training, I kinda knew what it was like [being a doctor], but I think it’s the repetitiveness, focusing on numbers and having to do it so many times a day, I mean I wince at the lancet, when it goes off as its getting to the point where it’s actually getting, you know, traumatised by the pain that comes from the pricked fingers” (Christina 15A).*

### Behavioural regulation

‘Behavioural regulation’ refers to a woman’s focus on self-monitoring effectively and planning how to incorporate this into her daily life. Women who decided to have all their testing and documentation equipment in a dedicated bag and leaving it at dedicated place at home indicated how helpful this was to undertake the testing regularly. Sharing their glycaemic target achievements on social media, with overseas GDM Facebook groups, and thinking of the health of their baby were identified as motivators to regulate behaviour.

*“You just put yourself into a routine. You just have to, for the baby, and have all your gear in a bag, ready to be used anytime and anywhere” (Sabrina 05).*

*“Yes, having it all planned helps. Every morning at 10.30 I have 30 minutes’ walk. And also after afternoon tea and dinner I have 30 minutes walking. It’s very good, and I feel I have more energy” (Casey 01A).*

### What affects a woman’s CBG concentration and how does she maintain optimal glycaemic control with this knowledge?

The themes emerging from the interviews from women with GDM relating to the second research question ‘What affects a woman’s CBG concentration and how does she maintain optimal CBG control with this knowledge?‘were categorised into five of the theoretical domains. These were: Knowledge, Beliefs about consequences, Environmental context and resources, Emotion, and Behavioural regulation (Table 5). The domains: Belief about consequences, Environmental context and resources and Behavioural regulation were represented most strongly in the context of this question.

### Knowledge

In the context of the second research question the domain ‘knowledge’ refers to a woman’s understanding of what affects her CBG concentrations. Women who knew the differences between carbohydrates, proteins, and fats, could read food labels and understood how exercises affected their blood glucose concentrations were more likely to embrace dietary and exercise changes and continue with regular blood glucose monitoring.

*“You just fill it up with other stuff, like veggies, depends on what you eat regularly, it you eat KFC all the time then your buggered” (Danielle 06).*

*“Yeah, so whenever I do my walking after meal, my blood sugar gets low right away, but if I snuggle in the bed after a meal, the blood sugar is high, that’s what I notice” (Belle 19A).*

Barriers identified included not knowing how to read food labels or how food intake and activity levels impact on glycaemic control or unable to read the information provided, as it was not in the woman’s first language. Two women knew how to increase their subcutaneous insulin, so they could continue eating their favourite sweets and carbohydrates and not be concerned about any behavioural lifestyle changes.

*“I don’t really understand what these food labels mean. I eat the same stuff anyway, not much use knowing it” (Jisha 04).*

*“So, I ask them, can I just have some insulin, and they say, "Okay". They give me the long-term insulin and now I can have sweets but my levels are ok” (Casey 01A).*

### Belief about consequences

The ‘Belief about consequences’ domain refers to a woman’s expectations about what affects her CBG concentration. Several women started eating the same food every day. Some women ordered weekly commercially available, pre-assembled ready for cooking, health food bags, as this enabled women to keep their CBG concentrations within the recommended glycaemic targets. While this method of food intake was identified as an enabler by the women, it is unclear how effective long-term lifestyle changes would be sustained, as the women were not planning to eat in a similar way after the baby was born. Further enablers were identified as hearing other women’s stories about GDM, which encouraged anticipated regret (‘I know if I do this I will regret it, therefore I will not do it’). This meant women were diligent about routinely exercising and following their diabetic diet.

*“I focused on it’s a short period of time, eating the same every day, you can get through it, and after pregnancy it’s going to be so awesome that you can eat what you want to eat, you focus on the fact that it’s not forever, I always think of trying to push a baby out that is too big, that’s an incentive, they can dislocate if it’s too wide, so I just focus on every little bit, makes a difference, that’s what I picked up from the obstetrician, you might go “oh this biscuit won’t hurt” but yeah it makes a difference, no option but do it consistently. I know of women who so regretted that they did not do it properly” (Annie 16A).*

Women in the study who believed that exercises had no effect on CBG concentration were not likely to engage in any physical activity. Women who believed that too much physical activity may cause pre-term labour would do occasionally a short walk. The belief that the diabetes medication would control CBG concentrations prevented women from engaging in understanding the effects of food intake and glycaemic control.

*“I’ve never tested after doing exercise, yeah, so I couldn’t say, I don’t belief it makes a difference, so don’t do it really” (Alice 10A).*

*“I don’t want my baby to come before 35 weeks, you know, I’m scared it comes early, more exercise makes it too early, but I will walk or swim after that time if it makes a difference” (Anna 07).*

*“I feel better now that I’m on the right medication. My sugars are well controlled and I don’t need to worry about eating and walking” (Erin 04B).*

### Environmental context and resources

In the context of this research question this domain refers to a woman’s access to food, exercise equipment and health professionals. Women in this study, who had access to group or individual sessions with a dietitian, could understand and alter their food intake and keep a food diary. Being taught CBG testing by a pharmacist or by a diabetes midwife in a group session was experienced as an enabler by most women interviewed. Having easy access to exercise equipment, such as a tread-mill or stationary bicycle, enabled women to exercise if they were unable to leave the house. Other enablers were identified as ensuring ‘right’ food in the pantry, having access to a vegetable garden, lower food costs, less fast food meals, and easy phone access to a diabetes dietitian.

*“Walking through a personalised diet is really helpful, and not just a mass-produced ‘try these things’ and straight access to the dietitian via phone or email. I know what I can and can’t eat now. Keeping a food diary has been good” (Anneri 14B).*

*“I bought a walker machine [treadmill] after being diagnosed with GDM to exercise. Every time I eat I do it. It is working well” (Belle 19A).*

Barriers were identified as lack of or limited access to, resources and health professionals. This included lack of access to transport to attend group or individual sessions with a dietitian, no considerations for ethnic food options, not being able to discuss the effects on glycaemic control in the woman’s first language, health professionals not looking at the food diary, higher food costs and unclear or no guidance about physical activities/exercises and its effect on CBG concentrations.

*“Not sure if some food puts it up, it’s possible, if I did a food diary I guess I could look it up, but that’s a bit tedious” (Toni 03).*

*“I didn’t read it, because it’s easier for him to read in English about what types of food you need to eat, it should be in colour and pictures. He doesn’t like to read either, but when you give me a colour picture, these are the things you need to eat a lot, and these are things you need to not eat in colour, that would make a difference, then I would understand” (Zeinab 12A).*

*“I don’t do much exercise because I am working all the time. Don’t know how to fit it in. Maybe someone needs sit down with me and show me how and when?” [to exercise] (Fiona 02A).*

### Emotions

The domain ‘Emotion’ refers to a woman’s reaction and feelings as to what affects her CBG concentrations. Enablers most commonly reported for were positive emotions, such as being happy and excited to understand the link between food intake and exercises and glycaemic control. Barriers most commonly reported related to negative emotions, such as being stressed about not seeing any difference in glycaemic control despite trying hard to follow the dietary guidelines and feeling hungry most of the time when trying to achieve optimal glycaemic control.

*“There are days when I am so worried that I am eating the wrong food and might hurt my baby, where I have checked myself 12 times just to see where I am staying at because the strict diet does not make a difference [to CBG concentrations], maybe I should just stop altogether? If you don’t know, you don’t know” (Aroha 10B).*

*“…but if I’m too hungry then I don’t care, which is quite often” (Elizabeth 08B).*

*“I was kind of worried about what the dietician was going to say because I did have a few highs like in my first week of trying and I remember just feeling so overwhelmed and walking in she said, ‘are you OK?’ and I just burst into tears, it was just one of those things. She said: Oh my goodness, I’m not going to tell you off or anything, we'll work through it” (Collette 09B).*

### Behavioural regulation

The domain ‘Behavioural regulation’ refers to a woman’s focus on self-monitoring effective food intake and exercise and planning how to incorporate this into daily life. Women who had action plans in place for physical activity and food intake, for example to do 20-min exercise after each main meal, bathing the toddler after the evening meal, playing ball games with the family, and starting a food and activity diary, found it easy to incorporate the changes into their daily life. Renaming exercise as physical activity and diet as food intake made a significant difference for women’s confidence level to self-monitor these.

*“Oh, you will laugh, but I don’t try to vacuum the floor, I brush the floor every night time on my knees with a brush and shovel, because I can’t go out and I get cold. My levels are good when I do this. No good levels when I do not do it” (Jisha 04A).*

Barriers to changing and regulating behaviour were mainly the dislike of having to exercise or to focus constantly on what to eat. Women noted that both stress and excitement would increase CBG concentrations and this discouraged effective self-monitoring.

*“For the baby shower, I was so good with the food but my levels were still high, it’s not just stress but also excitement that puts it up. So, what use is that then not to feel happy. May as well not do the testing” (Raman 17B).*

### What support have women found helpful/not helpful in learning about and maintaining CBG control?

The key themes emerging from the interviews for women with GDM relating to the third research question ‘What support have women found helpful/not helpful in learning about and maintaining CBG control?’ were categorised into four of the theoretical domains. These are: Beliefs about consequences, Reinforcement, Environmental context and resources and social influences (Table 6). The domains Environmental context and resources and Social influences were represented most strongly in the context of this question.

### Beliefs about consequences

The domain ‘Beliefs about consequences’ **r**efers to a woman’s expectation to sharing her diagnosis of GDM with others. Women shared their diagnosis and management of GDM with significant others and work colleagues when they believed this would gain them support for learning more about and maintaining optimal glycaemic control. Interestingly, when women perceived that sharing their diagnosis would generate a judgement and/or unhelpful advice they would not ‘tell’. Some women did not ‘tell’ because they felt protective towards their family members and did not want to worry them unnecessarily. This created a lonely place for some women.

*“It helps them to be more supportive if they know. I told them all. I don’t want them to bring sugary items when they visit” (Collette 09B).*

*“Did not tell, as I am big and people will say, ah, yes, you are fat, that did it” (Jean 16B).*

*“Did not tell parents and friends, as they get too worried, but a bit lonely and hard doing it without them” (Aliisa 02B).*

### Reinforcement

‘Reinforcement’ here refers to a woman’s ability to reinforce skills and coping strategies for self-support in maintaining optimal CBG control. Continuing with a written or creating a pictorial food diary (taking photos with a mobile phone camera), honestly and diligently documenting CBG results and rewarding glycaemic achievements with non-food items or activities (for example, going to the movies) were identified as enabling reinforcement of skills and coping strategies. Family activities such as family members guessing around the dinner table what the CBG levels will be before and after the meal, creating a graph for the fridge for charting CBG levels for all to view and using stickers to identify in the pantry/fridge which foods are healthy options for women with GDM to consume were further reinforcing enablers.

*“It’s kind of a fun time. My husband and my daughter guess what the number should be after I have done the pricking. If we are all right we reward us with going to the playground park with my daughter, she loves it and so do we” (BC 17A).*

### Environmental context and resources

The domain ‘Environmental context and resources’ refers to a woman’s ability to have access to learning resources and health professional services for optimal glycaemic control. Visual information, such as a food plate with portion sizes and access to informative websites about GDM were identified as enablers. Provision of a free health shuttle for clinic appointments, a hospital crèche for child care, an efficient appointment system reducing waiting times and partner and extended family welcomed at teaching sessions and clinic appointments contributed to women’s ability to perform CBG testing confidently and of feeling supported. Telephone access to discuss CBG results was available for most women and while only a few women used it, telephone access was considered a reassuring support. Provision of healthy food options by family and work colleagues was reported as a significant support.

*“Yeah husband attending info sessions was good but next time not during office time, evenings or weekends would be better. The food plate was very helpful, but maybe more Asian food on it would help too” (Amali 16).*

Several women had experienced barriers to accessing learning resources and health professional services for optimal glycaemic control. These included health professionals being impatient and inconsistent in their advice; not seeing the same health professional twice; not being taught how to check the glucometer; long waiting times at clinic appointments; not being taught in their first language, being unable to write into their food diaries in their first language; having no transport to attend teaching sessions or clinic appointments and poor parking facilities. Of the women who searched for information through Google, some became scared and would have preferred guidance to visit a website with clear and supportive information. Restaurants being unable to provide an ingredients list for meals on the menu was identified as another inaccessible resource. Provision of unhealthy food by family and work colleagues was reported as a significant barrier.

*“Google was a bit scary. So, it’s better just to stay away from it and get your questions answered at the clinic. But that google information was in Russian, and that was good. Yeah, they need to tell me where to look on the internet. Same with menus from restaurants, their ingredients could be listed on-line” (Lilly 18A).*

*“I saw a registrar who seemed very junior and gave me quite conflicting information to what everybody else had given me. So, I actually went back yesterday and saw a consultant, because I wasn’t happy. That improved things, but it took more time and to find a carpark is nearly impossible” (Erin 18B).*

*“He says, “Just eat whatever you want”, because he likes sweet stuff. Hard not to give in” (Tara 19B).*

### Social influences

The domain ‘Social influences’ refers to a woman’s access to social interaction to learning/reinforcing optimal blood glucose control. Some women joined an American Facebook group for women with GDM. While the glycaemic targets were different for the American counterparts, women in this study enjoyed swapping recipes, sharing tips about CBG testing, celebrating successes of achieving and maintaining glycaemic control and providing encouragement when glycaemic challenges were shared. Family and friend’s interest in all aspect of glycaemic control and meeting other women with GDM contributed to feeling supported and reinforcement for optimal glycaemic control.

*“So, I soon realised, after joining a [American] Facebook group, that most people struggle with cereals. So, I removed the cereal and just went to a two-egg breakfast, and that just evened it out. So, then I felt a bit better again” (Anneri 14B)*.

*“Yes, in the morning, if I want to sleep in then he will do for me the fingerpicks” (Shairin 11).*

Participating women identified social disconnections as barriers for learning and reinforcing optimal glycaemic control. This included unsupportive family members and workplaces, unavailability of a support group for women with GDM in New Zealand, on-line or face-to-face, being judged in public and being constantly’ told what to eat and what not to eat by family members.

*“…I guess that’s why I eat my chocolate with my yoghurt. I like chocolate, I’m going to have chocolate. You tell me I can’t, then I’m not going to listen. And I’m going to want it more and I’m going to binge eat it and don’t worry about my levels” (Aroha 10B).*

The results from the three questions explored in this study identified enablers and barriers for women with GDM representing their experiences of monitoring CBG concentrations, what affects this monitoring and what supports have been helpful for them to achieve optimal glycaemic control. As a summary, Table 7 outlines some considerations for practice and research that may be useful for health professionals and diabetes in pregnancy services providing care for women with GDM.

**Table 7:** Considerations for practice and research

Practice considerations	Research considerations
Monitoring for optimal glycaemic control	Monitoring for optimal glycaemic control
• Enable women with GDM to attend group teaching for CBG testing and interpretation and include women who have had GDM to share their stories. • Discuss individual strategies for regular CBG monitoring, food intake and physical activity. • Encourage partner and family attendance at any clinic or teaching sessions (may need to be offered at evenings or weekends). • Provide information relating to GDM in a woman’s first language and/or more visually, including ethnic food suggestions. • Investigate the possibility of community pharmacists’ involvement in teaching CBG testing.	• Explore opportunities for companies to create phone Apps, e.g. for electronic food and activity diaries, recording of CBG results and medication intake. • Do phone apps have an impact on optimal glycaemic control for women with GDM? • Does a name change for GDM reduce anxiety in pregnant women?
Dietary intake and exercise for glycaemic control	Dietary intake and exercise for glycaemic control
• Enable easy access to a diabetes dietitian with diet recommendations tailored to an individual woman’s context (cultural, financial, and emotional). • Engage in meaningful discussions about the content in a food diary and provide multi-modal opportunity for the woman to record the food diary in her first language or enable mobile phone photo collection of food intake. • Regularly address hunger for women with GDM. • Encourage a physical activity diary alongside the food diary. • Consider engaging a physical (activity) therapist for clear in-depth assessment and guidance of exercise that women can incorporate into their daily life.	• Does keeping a physical activity diary impact on glycaemic control? • Does engaging a physical activity therapist contributes to the understanding and up-take of physical activity for women with GDM? • Why do women with GDM seem to be hungry despite quality dietary recommendations? • What affect has self-imposed dietary practices by women with GDM during their pregnancy on long term lifestyle behaviour?
Support for optimal glycaemic control	Support for optimal glycaemic control
• Provide free CBG monitoring equipment, health shuttles and child care when attending clinic appointments and reduce clinic waiting times. • Consider face-to-face support groups for women with GDM. • Consider setting up a social media group for women. With current GDM (e.g. Facebook). • Include regular mental health assessment for women with GDM. • Provide direct phone access to multi-disciplinary health professionals.	• Limited research available for regular mental health assessment for women with GDM. • Limited research about the effect of a GDM diagnosis on partners and family members. • Limited research on how partners and families can best support a woman with GDM in their context. • Does social media or face-face group support make a difference for women with GDM for maintaining optimal glycaemic control?

## Discussion

Our results highlight the complex interactions between women with GDM monitoring their CBG concentrations, their understanding of the link between dietary intake, exercise and glycaemic control, having stress-free access to health care providers and resources, and their social context and support. The study used interviews and the validated TDF to determine the enablers and barriers women with GDM experience to achieve optimal CBG control. We categorised emerging enablers and barriers into a total of nine domains across three study questions. These were: Knowledge, Skills, Beliefs about capabilities, Beliefs about consequences, Reinforcement, Memory, attention and decision processes, Environmental context and resources, Social influences, Emotion and Behaviour regulation (Tables 4, 5 and 6). Through our iterative process we identified when no new themes were emerging within the TDF domains, thus ensuring that data saturation had been achieved [37, 38]. Transcript analysis revealed a range of enablers and barriers that impact on a woman’s ability to achieve optimal glycaemic control.

The initial response of women being diagnosed with GDM was predominantly of being shocked. At the time of the interview the women had generally accepted the diagnosis, knowing it would only last a finite time and were motivated by making a difference for the baby. Maternal shock, fear and anxiety associated with a diagnosis of GDM have been reported in the literature with a trend towards acceptance as the pregnancy progressed [39–44]. Kalra and colleagues [45] suggest that these findings support an onomastic (re-naming) opportunity, arguing that the phrase gestational diabetes can cause significant psychosocial morbidity. Alternative names suggested for GDM were Gestational Dysglycemia of Nutritional Origin (GDNO) or Pregnancy Related Intolerance to Glucose (PRIG). This indicates further research is needed to determine if an onomastic change would achieve less maternal psychosocial morbidity. Some women in our study, once they overcame the initial shock, thought that it was a ‘privilege ‘and a ‘good thing’ to have been diagnosed with GDM, as this supported change to a healthier lifestyle and provided them with skills such as being able to read and understand food labels. This advocates for an opportunity for promoting lasting lifestyle changes during the remainder of the pregnancy. Other studies reiterate these findings, and found that for some women with GDM, the knowledge gained enhanced the motivation and self-efficacy to initiate lasting lifestyle changes [40, 46, 47].

While most women accepted that they had GDM and adapted to the change, many women disliked the change of focus for their pregnancy to numbers of CBG concentrations, glycaemic targets, 90th percentile for fetal growth and maternal weight. This contributed to a feeling of reduced control, which exacerbated emotions and created barriers for some women. This meant a few women in the study did not continue with or reduced their self-monitoring of CBG concentrations, decided they were too busy to attend some of the clinic appointments, refused referrals for serial growth scans and were less committed to adhere to diet recommendations. Negative feelings acting as a deterrent to intervention up-take has been reported for women with GDM by some studies [41, 48, 49]. This suggests that emotional support and mental health assessments need to be an imperative part of heath care for women with GDM.

### Monitoring for optimal glycaemic control

Nearly all women in our study knew their optimal glycaemic targets and the importance to adhere to them. Despite this knowledge, participating women reported that this did not necessarily mean they would self-monitor their glycaemic control as advised. Responses varied on how they felt about their self-monitoring skills, if they had access to equipment, and their context, evident through the most strongly represented domains of Belief about capabilities, Emotions and Social influences. Women were less likely to do the CBG testing or stopped altogether for a variety of reasons. These included being scared and unsure about pricking their finger for CBG testing, playing a musical instrument, believing high CBG concentrations would harm their baby, being asked to leave a restaurant when testing, not being able to take a break to perform the test because of the nature of their work, and not being believed that their recorded CBG results were correct. Women were more likely to continue with regular CBG testing if they thought it was less painful than anticipated, attended a group session to learn how to perform CBG testing, took family members to teaching sessions, were shown by a community pharmacist how do to the testing, had the belief they knew how to do it, were praised by health professionals for their efforts, and had fun ‘pricking’ friends and family. There is a need for health professionals to provide clear and meaningful information about CBG testing, discuss strategies for overcoming barriers particularly in work situations, enable family members to be part of this process and to believe a woman’s CBG recordings (Table 7). These findings are echoed in other literature [41, 50]. The notion for a community pharmacist to initially teach women diagnosed with GDM how to perform CBG testing may be a valuable option to consider when time, cost and distance are a barrier. Some studies involving patients (pre-diabetic or with T2DM) self-monitoring their CBG concentration have found community pharmacies specialised in diabetes care can provide this service effectively [51, 52]. An extensive literature search did not identify any studies involving women with GDM and the effects of being taught CBG testing by a local community pharmacist. Clearly this is an area where further research is required (Table 7).

### Dietary intake and exercise for optimal glycaemic control

Our study demonstrated that the domains Belief about consequences, Environmental context and resources and Behavioural regulation were represented most strongly in the context of dietary intake and exercise for enabler and barrier identification. Studies have reported that women with GDM who were treated with dietary advice and were self-monitoring CBG concentrations had fewer macrosomic babies, less maternal weight gain and less birth trauma [8, 53]. A Cochrane systematic review assessed evidence from 19 trials for ten different dietary interventions and concluded that while dietary advice is the main strategy for managing GDM it remains unclear which type of diet is best [54]. Dietary self-management guided by CBG concentrations alone without a particular diet may be difficult for women with GDM. In our study, participants who understood the benefits and consequences of dietary self-management and regular exercise for controlling their CBG concentrations had access to professional dietetic advice and could incorporate effective physical activities into their daily life achieved optimal glycaemic control most of the time. This is consistent with other studies [44, 50, 55, 56]. Women in our study saw self-imposed dietary restrictions such as eating the same meal every day or ordering commercially pre-packed health food options as enablers, and for them these were solutions to their current hyperglycaemia, as GDM was understood to be transitory. This self-imposed practice resulted in a woman’s CBG concentrations staying within her recommended glycaemic targets most of the time. It is questionable if this approach would achieve long-term lifestyle changes. It is possible that the women in our study may not have understood the link between GDM and the risk for subsequent development of T2DM, and the importance of health behaviour regulation for reducing future diabetes risk [42, 57, 58]. Health professionals need to ascertain from women the reasons for any self-imposed dietary practices and ensure future health implications are understood. Further research is needed to explore in depth if self-imposed dietary practices by women with GDM during their pregnancy affect long term lifestyle behaviour (Table 7).

Over half of the women in this study identified a barrier to written information, as it was only provided in English. They wanted the health information in either their first language or for it to be more visually presented to better understand their GDM diagnosis, what optimal blood glucose control meant and to include ethnic food options (Table 7). These are similar findings reported by qualitative studies for women with GDM in Vietnam [42], Italy [59] and South Tamil Nadu [60]. Women in our study identified Google as a helpful tool, especially if they could access websites in their first language through Google. Health professionals need to be aware that women will access information beyond the clinic environment and that the quality of this information may vary. Health literacy providing clear and relevant health messages for women with GDM or other types of diabetes has been identified as an effective way to help people manage their own health care [61–63].

Most women commented on being hungry, but felt they could endure this for their babies’ health, if it kept their CBG concentrations within the recommended glycaemic targets. Dietetic advice needs to include how to address hunger for women with GDM (Table 7).

Regular aerobic exercises involving large muscle groups such as walking, swimming and stationary cycling have been reported to be beneficial in pregnancy and are not associated with harms to the baby [64, 65]. The prevalence of exercise for women with GDM during their pregnancy appeared to be related to their understanding of what type of exercise they could do and its duration. This was further compounded by their inability to incorporate exercises into their busy daily life and the fact that it was called exercise. The lack of specific recommendation on type, intensity, and duration of exercises from health professionals and women’s beliefs that exercises could cause pre-term labour or that rest is required when pregnant has been reported in the literature [48, 55, 66, 67]. Participating women, who approached exercises as meaning to be physical activity, were more likely to think outside the (exercise) square, and welcomed discovering which physical activity, such as bathing the toddler after the evening meal, had an impact on their glycaemic control. A Cochrane systematic review on exercise for pregnant women with GDM for improving maternal and fetal outcomes summarised evidence from 11 randomised controlled trials and found while exercises appeared to lower fasting and post-prandial CBG concentrations, they did not find any differences in other outcomes for pregnant women with GDM [68]. However, even if exercise does not provide any benefit during pregnancy, this change in lifestyle may persist after birth, and may help prevent the onset of type 2 diabetes and its long-term complications. A prospective study of 4554 women with previous GDM, who were followed for 16 years showed that increased physical activity levels lowered T2DM development and its risks [69]. This may mean that for women with GDM it could be worthwhile to record physical activities alongside or as part of their food diary for them to understand the effect physical activities have on their CBG concentrations (Table 7). It is common practice for the dietitian or the Lead Maternity Carer (LMC) midwife to recommended daily walking but without further in-depth guidance. Meeting with a physical activity professional or therapist who assesses where and what physical activities could be adapted to a woman’s daily context, alongside other health professionals at the diabetes in pregnancy clinic, may be an option to consider and would benefit from further research (Table 7).

### Support for glycaemic optimal control

Women reported that support from partners, family, friends, work colleagues and health professionals made a significant difference for them to accept their diagnosis, adhere to prescribed treatment and maintain optimal glycaemic control. This support facilitated self-management and healthy lifestyle behaviours. Partners and extended family support was reported as valuable in particular for increasing exercise and the provision of healthy meals. Similar findings have been reported in the literature [39, 41, 46, 50]. The key domains identified for this section of social influences and belief about consequences reflect this. Other suggestions for support included joining a social media network for women with current GDM, for example on Facebook, and/or attend a local support group for women with GDM. Neither of these are currently available in New Zealand and support organisations for Diabetes or DHB’s may want to consider this (Table 7).

Some women in this study found their family’s excessive concerns or providing unhealthy meals a challenge and reported that this contributed to them feeling stressed and unable to perform CBG monitoring. A qualitative study of perceived needs in women with GDM found similar findings and indicates the importance for health professionals to increase their awareness for the need of social support for women with GDM [70]. Other studies including women with borderline GDM and T2DM reiterate this [54, 71–73] and recommend, that health professionals as part of clinic appointments need to include discussions about effective strategies to cope with situations that are challenging for women with GDM. Research about the effect of a GDM diagnosis on partners and family members and how they can best support a woman with GDM in their context is limited (Table 7). Complexities of social determinants of health is often studied with ethnographic research [74] and it may be appropriate to encourage this type of research for partner and family experience who are living and supporting women with GDM.

Within the identified key domain of belief about consequences, a surprise finding was that several women reported not sharing their GDM diagnosis with anyone other than their partners. The main reasons for this decision was fear of being judged, not wanting to be scrutinised for daily activities including food intake, or not wanting to worry extended family members. This created social isolation, and contributed to a woman’s feeling of shame, guilt, and reduced her ability to achieve optimal glycaemic control. Qualitative studies support these findings [43, 75–77]. The women in our study had not shared this decision with their respective health professionals. This suggests the need for greater awareness among health professionals that some women with GDM ‘do not tell’ and on-going assessment of a woman’s mental well-being should be included in the health services provision [40].

This study adds to the body of knowledge about enabling women with GDM to achieve optimal glycaemic control. While some studies have explored the GDM experience from the woman’s point of view, none have specifically studied the enablers and barriers to achieving optimal glycaemic control using the validated Theoretical Domains Framework. The sample size was reflective of a cross section of the demographics of New Zealand’s pregnant population and reached data saturation.

Limitations of our study were that participating women were not from rural or remote areas in New Zealand and only women who were fluent in English were eligible. Women from different cultural backgrounds were well represented in this study (Table 3). It is unclear if the interviews with women in their first language would have elicited different enablers and barriers for optimal glycaemic control. Women interviewed often asked for information in their first language. Future research should consider conducting interviews in a participant’s first language.

## Conclusions

This qualitative study categorised identified enablers and barriers for women with GDM to achieve optimal glycaemic control into 10 theoretical domains across three main areas. This provided insights to how women adapt to regular CBG self-monitoring, adhere to recommended treatments, undertake necessary lifestyle changes and can be supported.

Barriers included: lack of health information, teaching sessions, consultations, and food diaries in a woman’s first language; long waiting times at clinic appointments; seeing a different health professional every clinic visit; inconsistent advice; no tailored physical activities assessments; not knowing where to access appropriate information on the internet; unsupportive partners, families, and workplaces; and unavailability of social media or support groups for women with GDM. Perceived judgment by others led some women only to share their GDM diagnosis with their partners. This created social isolation.

Enablers included: the ability to attend group teaching sessions with family and hear from women who have had GDM; easy access to a diabetes dietitian with diet recommendations tailored to a woman’s context including ethnic food and financial considerations; free CBG monitoring equipment, health shuttles to take women to appointments; child care when attending clinic appointments; and being taught CBG testing by a community pharmacist.

The enablers and barriers identified are multidimensional and may assist health professionals and diabetes in pregnancy services on how best to meet the needs of this diverse group of women and their families to achieve optimal CBG control and so reduce adverse outcomes for women with GDM and their babies.

## Abbreviations

BMI: Body Mass Index
CBG: Capillary Blood Glucose
DHB: District Health Board
GDM: Gestational Diabetes Mellitus
LMC: Lead Maternity Carer
T2DM: Type 2 Diabetes Mellitus
TDF: Theoretical Domains Framework
WHO: World Health Organisation.
